# Non-invasive assessment of hepatic mitochondrial metabolism by positional isotopomer NMR tracer analysis (PINTA)

**DOI:** 10.1038/s41467-017-01143-w

**Published:** 2017-10-06

**Authors:** Rachel J. Perry, Liang Peng, Gary W. Cline, Gina M. Butrico, Yongliang Wang, Xian-Man Zhang, Douglas L. Rothman, Kitt Falk Petersen, Gerald I. Shulman

**Affiliations:** 1 Departments of Internal Medicine, Yale University School of Medicine, New Haven, CT 06520 USA; 2 Departments of Radiology and Biomedical Imaging, Yale University School of Medicine, New Haven, CT 06520 USA; 3 Departments of Biomedical Engineering, Yale University School of Medicine, New Haven, CT 06520 USA; 4 Departments of Cellular and Molecular Physiology, Yale University School of Medicine, New Haven, CT 06520 USA; 5 Departments of Howard Hughes Medical Institute, Yale University School of Medicine, New Haven, CT 06520 USA

## Abstract

Hepatic mitochondria play a central role in the regulation of intermediary metabolism and maintenance of normoglycemia, and there is great interest in assessing rates of hepatic mitochondrial citrate synthase flux (*V*_CS_) and pyruvate carboxylase flux (*V*_PC_) in vivo. Here, we show that a positional isotopomer NMR tracer analysis (PINTA) method can be used to non-invasively assess rates of *V*_CS_ and *V*_PC_ fluxes using a combined NMR/gas chromatography-mass spectrometry analysis of plasma following infusion of [3-^13^C]lactate and glucose tracer. PINTA measures *V*_CS_ and *V*_PC_ fluxes over a wide range of physiological conditions with minimal pyruvate cycling and detects increased hepatic *V*_CS_ following treatment with a liver-targeted mitochondrial uncoupler. Finally, validation studies in humans demonstrate that the *V*_PC_/*V*_CS_ ratio measured by PINTA is similar to that determined by in vivo NMR spectroscopy. This method will provide investigators with a relatively simple tool to non-invasively examine the role of altered hepatic mitochondrial metabolism.

## Abstract (web summary)

Liver mitochondrial metabolism plays an important role for glucose and lipid homeostasis and its alterations contribute to metabolic disorders, including fatty liver and diabetes. Here Perry et al. develop a method for the measurement of hepatic fluxes by using lactate and glucose tracers in combination with NMR spectroscopy.

## Introduction

Hepatic mitochondrial function plays a critical role in the regulation of liver and whole-body glucose and fat metabolism and there is great interest in understanding the potential role for alterations in hepatic mitochondrial activity in the pathogenesis of non-alcoholic fatty liver disease (NAFLD), non-alcoholic steatohepatitis (NASH) and type 2 diabetes (T2D) as well for the evaluation of potential novel therapies targeting hepatic mitochondrial fat oxidation to treat these diseases. Although recent studies by our group have demonstrated the utility of in vivo ^13^C magnetic resonance spectroscopy (MRS) to directly assess rates of hepatic mitochondrial oxidation flux (*V*_CS_) and pyruvate carboxylase flux (*V*_PC_) in humans^1, 2^, application of this method is expensive, time- and labor-intensive, and requires an in vivo wide-bore (>0.8 m), high-field (≥4 Tesla) magnetic resonance imaging system modified to do ^13^C MRS, which is available at only a few academic medical centers worldwide. To date the only non-invasive tracer method that has been described to assess hepatic mitochondrial fluxes in vivo utilizes [^13^C_3_]propionate^3–6^. A disadvantage of the propionate method is that it can alter hepatic mitochondrial metabolism in part through generation of high concentrations of propionyl-CoA^7, 8^. A key motivation for this study is to develop an alternative non-invasive tracer method to model hepatic metabolism in vivo with minimal perturbation of hepatic mitochondrial metabolism.

To address this unmet need, we have developed a simple positional isotopomer Nuclear Magnetic Resonance (NMR) tracer analysis (PINTA) method by which hepatic glucose production, anaplerosis and citrate synthase flux can be non-invasively assessed based on NMR and gas chromatography/mass spectrometry (GC/MS) analysis of plasma following an infusion of [3-^13^C]lactate (Supplementary Fig. 1a, b). To validate this method, we performed independent cross-validation studies and observed an excellent correlation (*P* < 10^−15^, *R*^2^ = 0.99) between *V*_PC_/*V*_CS_ calculated using PINTA and our previous ex vivo NMR tracer technique in rats^9, 10^, and similar *V*_PC_/*V*_CS_ ratios measured in healthy human subjects using two independent tracer methods (infusion of [3-^13^C]lactate or [1-^13^C]acetate^1, 2^). Validation studies demonstrate the ability of the PINTA method to measure an increase in *V*_CS_ flux following treatment with a controlled-release mitochondrial protonophore (CRMP), which promoted liver-targeted mitochondrial uncoupling^11^ and an increase in the *V*_PC_/*V*_EGP_ ratio after an extended fast. Taken together these data demonstrate that the PINTA method is sufficiently sensitive to demonstrate meaningful differences in hepatic mitochondrial flux rates in various physiological and pathophysiological states in both humans and rodent models of diabetes, NAFLD/NASH and other metabolic diseases. Furthermore, this method will be very useful for assessing target engagement for novel therapies that are currently being developed to promote increased hepatic mitochondrial fatty acid oxidation for the treatment of NAFLD/NASH and T2D.

## Results

### *V*_PC_/*V*_CS_ and *V*_PC_/*V*_EGP_ are similar by PINTA and ex vivo NMR

**Fig. 1:**
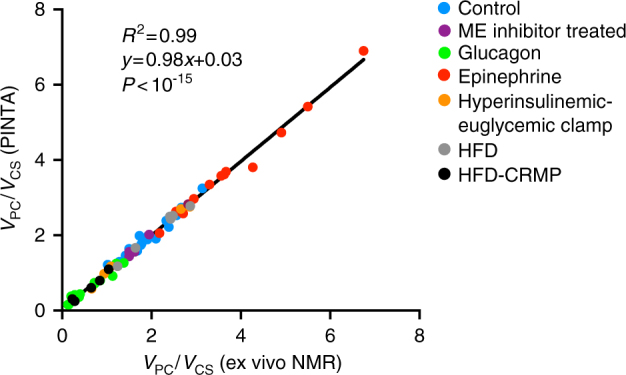
*V*_PC_/*V*_CS_ ratios measured by PINTA and ex vivo NMR. This ratio is identical whether measured by PINTA or ex vivo NMR analysis of rat livers

**Fig. 2:**
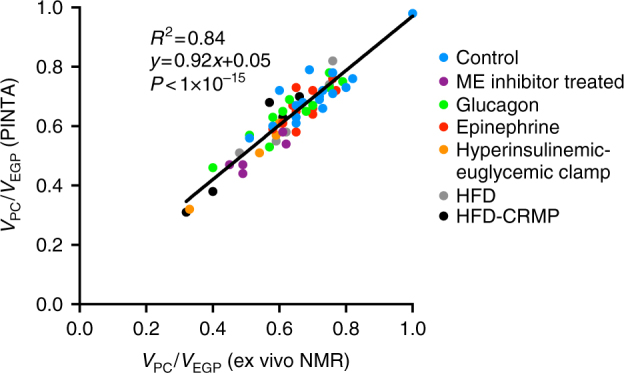
*V*_PC_/*V*_EGP_ ratios measured by PINTA and ex vivo NMR. This ratio correlates tightly when measured by PINTA with the *V*_PC_/*V*_EGP_ ratio determined using ex vivo NMR analysis

Analysis of plasma lactate and glucose enrichment in the rat studies confirmed unchanged plasma lactate concentrations and steady-state enrichment after 2 h of isotope infusion (Supplementary Fig. 2a, b). The hepatic glucose and glutamate data obtained from all rats in this study are shown in Supplementary Tables 1 and 2. Linear regression analysis demonstrated a strong correlation between the hepatic *V*_PC_/*V*_CS_ ratio measured with PINTA and that generated with our previously established ex vivo NMR method^1, 10^ (*R*^2^ = 0.99, slope = 0.98) in seven different groups of rats: (1) chow-fed rats; (2) high-fat-fed rats; (3) rats undergoing a hyperinsulinemic-euglycemic clamp to mimic postprandial hyperinsulinemia, and rats treated with: (4) glucagon; (5) epinephrine; (6) malic enzyme (ME) inhibitor; and (7) CRMP to promote increased hepatic mitochondrial oxidation (Fig. 1). We next compared the correlation between *V*_PC_/*V*_EGP_ ratios measured with ex vivo and PINTA analysis and confirmed a strong linear relationship between the two ratios (R^2^ = 0.84, slope = 0.92) (Fig. 2).

### Plasma can be used for PINTA analysis

**Fig. 3:**
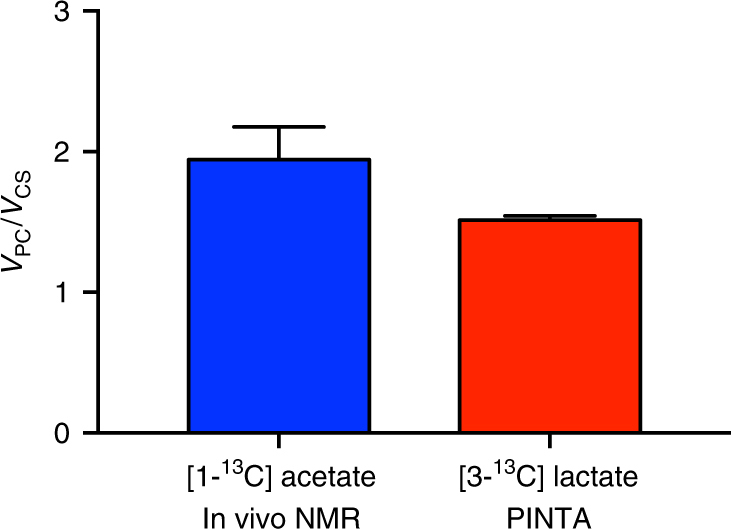
*V*_PC_/*V*_CS_ ratios in healthy human control subjects. The measured ratios are comparable as measured by in vivo NMR at 4 T or by PINTA. Data are presented as the mean ± S.E.M. of *n* = 3

Next, in order to investigate whether this method could be applied to non-invasively measure hepatic fluxes in humans, we first confirmed a strong correlation between liver and plasma glucose enrichment in rat samples (Supplementary Fig. 2c, d), demonstrating that the plasma glucose enrichment can be used as a surrogate for hepatic glucose enrichment in cases where non-invasive measurement of fluxes is of particular importance. We then measured the *V*_PC_/*V*_CS_ ratio in three healthy human subjects following a 12 h overnight fast, and found that the two methods (in vivo NMR analysis of positional glutamate enrichment^1^ or PINTA analysis of plasma glucose) gave similar results, with *V*_PC_/*V*_CS_ ~ 1.5–1.9 using both methods (Fig. 3, Supplementary Fig. 3a, b). The *V*_PC_/*V*_EGP_ ratio was 0.50 ± 0.02, consistent with previous measurements of gluconeogenic contributions to endogenous glucose production in humans following an overnight fast^12^.

### PINTA yields expected changes in fluxes with known perturbations

**Fig. 4:**
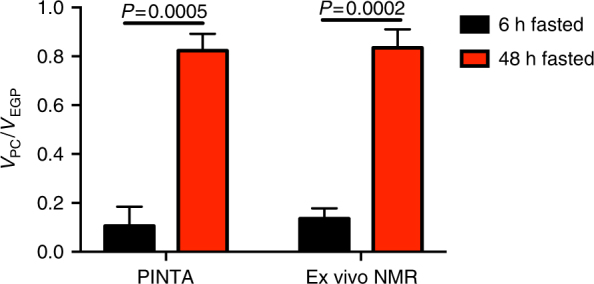
*V*_PC_/*V*_EGP_ increases with an extended fast. The *V*_PC_/*V*_EGP_ ratios measured using the two techniques are identical in both 6 and 48 h fasted rats. Data are the mean ± S.E.M. of *n* = 4 rats per time point

Next, to validate that the PINTA method is sensitive enough to detect expected differences in fluxes with alterations in hepatic mitochondrial oxidation we assessed the effect of a controlled-release mitochondrial protonophore, which we have previously demonstrated safely promotes liver-targeted mitochondrial uncoupling in vivo^11^, and found that this intervention increased *V*_CS_ flux two-fold and suppressed both EGP and *V*_PC_ fluxes (Table 1). We then measured the *V*_PC_/*V*_EGP_ ratio in glycogen-depleted rats fasted for 48 h and compared the *V*_PC_/*V*_EGP_ flux ratio to rats that had fasted for just 6 h, after a prior fast and refeeding to increase liver glycogen content similar to the recently-fed state (Supplementary Fig. 4). PINTA measurements demonstrated that *V*_PC_/*V*_EGP_ increased six-fold in hepatic glycogen-depleted rats fasted for 48 h, with the percentage of glucose production from pyruvate carboxylase flux increasing from ~13 to ~80% (Fig. 4).

**Table 1:** PINTA detects increased *V*_CS_ flux in high-fat-fed rats treated with a liver-targeted mitochondrial uncoupler

**Parameter**	**Control**	**CRMP**
*V*_PC_/*V*_CS_	1.50 ± 27	0.48 ± 0.12**
*V*_PC_/*V*_EGP_	0.79 ± 0.05	0.60 ± 0.06*
EGP (μmol/(kg BW-min))	39 ± 1	33 ± 1**
*V*_PC_ (μmol/(kg BW-min))	31 ± 3	20 ± 2**
*V*_CS_ (μmol/(kg BW-min))	23 ± 4	49 ± 9*

*V*_PC_/*V*_CS_ and *V*_PC_/*V*_EGP_ ratios, as well as absolute rates of endogenous glucose production (EGP), *V*_PC_, and *V*_CS_ flux were all measured using PINTA analysis of livers from rats infused with [3-^13^C]lactate. *P < 0.05, **P < 0.01 by the two-tailed unpaired Student’s *t*-test. Data are the mean ± S.E.M. of *n* = 5–6

## Discussion

Alterations in hepatic fatty acid metabolism play a critical role in the pathogenesis of hepatic insulin resistance and increased rates of gluconeogenesis in patients with T2D^13–15^ and alterations in hepatic mitochondrial metabolism have been proposed to play a key role in the pathogenesis of NAFLD^6, 16, 17^ and NASH^18^. Furthermore liver-targeted agents to promote increased hepatic fatty acid oxidation through inhibition of acetyl-CoA carboxylase^19, 20^ and liver-targeted^11, 21, 22^ and untargeted^23^ mitochondrial uncoupling agents are currently being developed to treat NAFLD/NASH and T2D. Thus there is great interest in developing non-invasive methods to assess rates of hepatic mitochondrial fatty acid oxidation and gluconeogenesis in vivo. Here we show that by simply assessing the positional isotopomer enrichments of the C4 and C5 carbons of plasma glucose by a combined ^13^C NMR/GC-MS method following an infusion of [3-^13^C]lactate we can non-invasively assess rates of hepatic mitochondrial oxidative (*V*_CS_) and gluconeogenic fluxes from pyruvate (*V*_PC_) in awake rodents and humans. Furthermore, by combining these *V*_PC_/*V*_CS_ relative flux measurements with assessment of endogenous glucose production and assessment of the ratio of *V*_PC_/*V*_EGP_ enrichment calculated from the m + 1 and m + 2 enrichments of glucose, we can estimate absolute rates of *V*_CS_ flux and *V*_PC_ flux.

To validate this method we first measured the ratio of hepatic pyruvate carboxylase flux to hepatic citrate synthase flux (*V*_PC_/*V*_CS_) using two methods following a steady-state infusion of [3-^13^C]lactate and [3-^3^H]glucose, comparing data obtained using our previously established ex vivo NMR analysis^1, 7, 11^ to the newly developed PINTA analysis (Supplementary Tables 1 and 2) in seven groups of rats undergoing various physiological perturbations which could alter hepatic mitochondrial activity: (1) control chow-fed; (2) insulin-resistant high-fat-fed; (3) hyperinsulinemic-euglycemic clamped; (4) glucagon infused; (5) epinephrine-infused; (6) ME inhibitor^7^ treated; and (7) liver-targeted mitochondrial uncoupler (CRMP^11^) treated rats. Using this approach, we observed a 1:1 correlation (Fig. 1; *R*^2^ = 0.99, slope = 0.98) for *V*_PC_/*V*_CS_ using these two methods. Both methods rely on a similar small set of assumptions, including having reached steady state in the metabolites of interest and negligible enrichment in [1-^13^C]pyruvate, [1-^13^C]alanine, and [1-^13^C]acetyl-CoA. We have validated these assumptions in the current study by demonstrating stability of glucose enrichment in both rats and humans (Supplementary Figs. 2b and 3a) and confirming minimal hepatic bicarbonate enrichment (1.5 ± 0.1%).

Next, we used our PINTA and ex vivo NMR methods to calculate the ratio of *V*_PC_/*V*_EGP_. We observed a strong correlation (Fig. 2; *R*^2^ = 0.84, slope = 0.92) between ratios calculated using the two methods in all groups of rats. The calculation of this flux requires a different but related set of assumptions. PINTA measurement of *V*_PC_/*V*_EGP_ assumes, in addition to achievement of steady state, a binomial probability analysis describing the distribution of ^13^C-glucose isotopomers resulting from the synthesis of two ^13^C-labeled trioses, and the presence of just one gluconeogenic precursor pool enrichment. The ex vivo NMR method, as we have described and validated^10^, assumes the presence of rapidly equilibrating small pools in the TCA cycle that are unperturbed by the tracer^24^, complete equilibration of C2 and C3 OAA^25^, and that the enrichment of malate reflects that of OAA. The strong correlation between two methods with differing assumptions is reassuring regarding the applicability of each method; however, future studies—ideally comparing PINTA with [3-^13^C]lactate tracer to an alternative stable isotope tracer—will be required to test the utility of PINTA in additional settings. Taken together these data demonstrate that the analysis of glucose by the PINTA method (Supplementary Tables 1 and 2) closely approximates the *V*_PC_/*V*_CS_ and *V*_PC_/*V*_EGP_ ratios measured using an invasive method that requires rapidly snap freezing 3–4 g of liver tissue in rodents within 10 s of intravenously administered anesthesia.

The primary goal of this report is to present a non-invasive method to measure hepatic oxidative and gluconeogenic fluxes that can be applied to humans. To examine the potential of PINTA to serve this purpose, we used two independent and complementary methods to calculate the ratio of *V*_PC_/*V*_CS_ in humans infused with [1-^13^C]acetate and [3-^13^C]lactate in separate studies. We have previously reported extensive validation studies to support the use of this method for measuring hepatic fluxes in vivo^1, 2^ and now demonstrate that PINTA measures the ratio *V*_PC_/*V*_CS_ similar to that determined using in vivo NMR analysis.

Next, in order to confirm the sensitivity of PINTA measurements to expected changes in mitochondrial flux rates, we performed a validation study utilizing treatment with a liver-targeted mitochondrial uncoupling agent, CRMP, which we have previously established can selectively increase rates of hepatic mitochondrial oxidation in vivo without inducing hyperthermia or any associated hepatic or systemic toxicities^11, 21, 26, 27^. As compared to vehicle-treated control rats, animals treated with CRMP exhibited a 2.5-fold increase in hepatic *V*_CS_ flux consistent with its effects to increase hepatic tricarboxylic acid cycle activity through uncoupling of mitochondrial oxidative-phosphorylation activity. This 2.5-fold increase in rates of hepatic mitochondrial oxidation induced by CRMP was associated with a ~70% reduction in the *V*_PC_/*V*_CS_ ratio and ~10 and ~30% reductions in rates of endogenous glucose production and *V*_PC_ flux, respectively (Table 1). Finally, we examined *V*_PC_/*V*_EGP_ flux in glycogen-replete rats fasted for 6 h and compared this ratio to glycogen-depleted rats fasted for 48 h. An extended fast increased *V*_PC_/*V*_EGP_ flux six-fold, consistent with a shift in reliance upon increased mitochondrial pyruvate carboxylase flux for glucose production in the absence of hepatic glycogen. These data demonstrate that the PINTA method is sufficiently sensitive to detect expected differences in both relative and absolute *V*_PC_/*V*_CS_ fluxes as well as in the ratio *V*_PC_/*V*_EGP_ with previously characterized physiological perturbations.

The selection of isotope is always a critical factor in any tracer metabolic study and [3-^13^C]lactate has many advantages over other stable isotopes that have been used to assess hepatic mitochondrial and glucose metabolism. First, plasma lactate concentrations are 10–100 fold greater than the plasma concentrations of glycerol, acetate or propionate and therefore significant ^13^C enrichment in hepatic lactate and its intraheptocellular metabolites can be achieved without significantly perturbing whole-body lactate metabolism. Furthermore, compared to glycerol, acetate and propionate, lactate has the lowest periportal perivenous gradient across the liver bed and therefore hepatocellular flux determinations using this isotope will be least impacted by any heterogeneity of mixing of tracer between periportal and perivenous hepatocytes^28–30^. In addition, [U-^13^C]propionate, when administered at doses commonly used to trace hepatic mitochondrial metabolism in human and animal studies, markedly increases plasma propionate concentrations, hepatic TCA cycle intermediates and hepatocellular propionyl CoA concentrations, a potent allosteric activator of pyruvate carboxylase leading to profound alterations in hepatic mitochondrial fluxes^7^. In contrast [3-^13^C]lactate can be infused at a rate that provides sufficient label to measure hepatic mitochondrial fluxes without significantly altering hepatocellular mitochondrial metabolites/fluxes^7, 8^. Because of its reliance on the assumption that pyruvate cycling is minimal the PINTA method would be of limited utility in settings where pyruvate kinase (PK) and/or ME flux is high relative to pyruvate carboxylase flux. To address this assumption, we have previously assessed rates of *V*_PK+ME_ flux and found these rates to be very low compared to rates of *V*_PC+PDH_ flux in healthy control subjects and insulin-resistant subjects with NAFLD following an overnight fast^2^. Consistent with these prior studies, we found in this study that *V*_PK+ME_ flux relative to *V*_PC+PDH_ flux is less than 6% in overnight fasted rodents (Supplementary Table 1), which we find using a complete equation including these fluxes will have minimal impact on our estimates of *V*_PC_ and *V*_CS_ fluxes. This finding makes teleological sense in that rates of hepatic glycolysis (*V*_PK_) would be expected to be minimal relative to rates of *V*_PC_ flux under fasting conditions in order to provide maximal net flux through gluconeogenesis to support obligate glucose-utilizing organs like the brain and renal medulla while minimizing energy dissipation due to futile cycling. However, under other conditions such as hyperthyroidism, which significantly increases rates of hepatic pyruvate cycling due to increased *V*_ME_ flux^31, 32^, as well as under hyperglycemic-hyperinsulinemic conditions, when rates of hepatic glycolysis would be expected to be significantly increased, it will be necessary to adjust these PINTA determined rates of *V*_PC_/*V*_CS_ flux using the equation provided in the Methods.

The use of the PINTA method to measure *V*_PC_ flux also has advantages over the ^2^H_2_O method^33–36^ to measure gluconeogenic flux rates. In addition to the improved specificity afforded by measuring *V*_PC_ instead of total gluconeogenesis, infusion of [3-^13^C]lactate is practically simpler for both investigator and subject: the ^2^H_2_O method requires dosing of ^2^H_2_O several hours prior to assessment of gluconeogenesis in order to allow adequate time for ^2^H_2_O equilibration in the whole-body water space. Furthermore, ^2^H_2_O administration is often associated with dizziness, nystagmus, nausea and occasional vomiting, which can be a limiting factor in the clinical setting.

In summary, here we describe a non-invasive (PINTA) method to measure rates of hepatic mitochondrial oxidation and pyruvate carboxylase flux in vivo during an infusion of [3-^13^C]lactate in combination with a glucose tracer to assess rates of endogenous glucose production. It is anticipated that this method will provide investigators with a relatively simple and widely available means of examining the role of altered hepatic mitochondrial and glucose metabolism in various physiologic and pathophysiologic states in humans and rodent models of diabetes as well as to examine target engagement for novel therapies that are currently being developed to promote increased rates of hepatic fatty acid oxidation for the treatment of NASH and T2D.

## Methods

### Animal studies

All experiments were approved by the Yale University Institutional Animal Care and Use Committee. Male Sprague-Dawley rats (~10 weeks of age) were ordered from Charles River (Wilmington, MA) at ~300 g and fed regular chow unless otherwise specified. High-fat-fed rats ordered at the same age and weight were given ad lib access to a safflower oil-based high-fat diet (Dyets, Bethlehem, PA, #112245) for 4 weeks, the latter 2 weeks of which they were treated with either a controlled-release mitochondrial protonophore^11^ (1 mg/kg in a small amount (~200 mg) of peanut butter) or the same amount of peanut butter vehicle each day. Before studies, rats underwent surgery under general isoflurane anesthesia to place polyethylene catheters in the jugular vein and common carotid artery (PE90 and PE50 tubing, respectively; Instech, Plymouth Meeting, PA). After 1 week, recovery was verified by confirming that the rats had regained their pre-surgical weight. Unless otherwise specified, all experiments were performed following an overnight fast (16 h food withdrawal before euthanasia). In both the rodent and human studies, data are represented as the mean of two technical replicates for each subject (i.e., two technical replicates per biological replicate). Sample sizes were selected to give power to detect moderate to large differences between groups (*n* = 5–6 per group), and all animals were included in the final analysis.

### Human studies

All protocols were approved by the Yale University Human Investigation Committee, and written informed consent was obtained from each participant after explanation of the purpose, nature, and potential complications of the study. We studied three healthy, normal weight men (<4% hepatic triglyceride); this sample size was chosen to give sufficient power to detect large differences between groups and to demonstrate the practical feasibility of this tracer method.

### Tracer studies

All rats were infused with [3-^13^C]lactate (Sigma, St. Louis, MO; 120 μmol/(kg-min) prime for 5 min, 40 μmol/(kg-min) continuous infusion) and [3-^3^H]glucose (PerkinElmer, Waltham, MA; 0.45 μCi/(kg-min) prime for 5 min, 0.15 μCi/(kg-min) for 120 min. Certain rats were treated with interventions (ME inhibitor, hydroxymalonate, Sigma, 200 mg/kg by IP injection at time zero of the tracer infusion; glucagon, Sigma, 5 ng/(kg-min) by continuous intra-arterial infusion throughout the tracer infusion; epinephrine, Sigma, 2 μg/(kg-min) by continuous intra-arterial infusion throughout the tracer infusion; and hyperinsulinemic-euglycemic clamp: insulin prime 40 mU/kg at time zero of the clamp and 4 mU/(kg-min) infusion throughout the tracer infusion, with a variable glucose infusion rate infused to maintain euglycemia ~100–110 mg/dl) as previously described^7^ following randomization to groups using a random number generator. The investigator performing the animal studies was not blinded for practical reasons, but the investigators analyzing fluxes were blinded as to the group allocation. Some of the rats utilized in this study (control, ME inhibitor, glucagon, epinephrine and clamp) have yielded previously published data (EGP, *V*_PK+ME_/(*V*_PC+PDH_))^7^; however, the fluxes presented in this study have not been analyzed or reported previously.

In the human studies, each subject participated in two studies, each following a 12 h overnight fast, and received an IV infusion of [1-^13^C]acetate (13 μmol/(kg-min)) or [3-^13^C]lactate (36.1 μmol/(kg-min)) on separate occasions^2, 37^. *V*_PC_/*V*_CS_ was determined by measuring the rate of incorporation of [1-^13^C]acetate into [1-^13^C] and [5-^13^C]glutamate using in vivo MRS^2^ during the [1-^13^C]acetate infusions, and by PINTA analysis of a plasma sample obtained at steady-state in the [3-^13^C]lactate infusions. The *V*_PC_/*V*_EGP_ ratio was measured by PINTA analysis of steady-state plasma after a [3-^13^C]lactate infusion. The *V*_PC_/*V*_CS_ ratio from these subjects has been published as a subset of the data included in our previous paper^2^; however, the PINTA analysis of these subjects has not been performed or reported previously.

### Flux modeling: PINTA

Our flux calculations require the following equations using [^13^C] glucose positional enrichment in plasma or liver, determined by NMR and GC/MS mass spectrometry as described below. The labeling patterns associated with the fluxes and the relevant pathways are shown in Supplementary Fig. 1. *V*_PC_ is the rate of pyruvate carboxylase flux as determined from the anaplerotic flow into OAA. *V*_CS_ is defined as the flux of the citrate synthase reaction in the citric acid cycle. As discussed previously the calculated anaplerotic flux can also include ME flux but under the conditions studied we have shown that ME flux is negligible compared to anaplerotic flux^2, 7^. Table 2 shows the equations used to calculate relative fluxes by PINTA.

**Table 2:** Flux modeling ratios (*V*_PC_/*V*_EGP,_ Eq. (1), and *V*_PC_/*V*_CS_, Eq. (2)) used in the PINTA method

**Ratio**	**Calculation**
$\frac{{{V_{{\rm{PC}}}}}}{{{V_{{\rm{CS}}}}}}$	$\left( {\frac{{\left[ {5 - {}^{13}C} \right]{\rm glucose}}}{{2*\left( {\left[ {4 - {}^{13}C} \right]{\rm glucose}} \right)}}} \right) - 1$ (1)
$\frac{{{V_{{\rm{PC}}}}}}{{{V_{{\rm{EGP}}}}}}$	$\frac{{\rm G2}}{{{\rm XFE^2}}}$ (2)
	With G2 and XFE as defined below

The derivations and further definitions of these equations are given below

### Calculation of *V*_PC_/*V*_CS_

The ratio of $\frac{{{V_{{\rm{PC}}}}}}{{{V_{{\rm{CS}}}}}}$ (Eq. (1)) was calculated as described below based on a model of hepatic metabolism developed by Katz for radiolabeled isotopes^38^. In this calculation, we assume that the flux from PC will continue through PEPCK and subsequent gluconeogenic reactions, and end up in liver glucose. We note that this will be a maximum estimate of the PC contribution due to the possibility of futile cycling where PEP is converted by PK into pyruvate. Our previous in vivo animal and human studies suggest that *V*_PK+ME_ flux is relatively low compared to *V*_PC_ flux under fasting conditions^2, 7^. Consistent with these prior studies we find that *V*_PK+ME_ flux/*V*_PC+PDH_ flux is relatively low (<15%) under several physiologic conditions (Supplementary Table 1) and that these low rates of pyruvate cycling will have a minimal impact of our estimated *V*_PC_/*V*_CS_ ratio.

In order to avoid any impact of pentose cycling on our estimate of the *V*_PC_/*V*_CS_ ratio we examine only the relative ^13^C labeling in carbons C4 and C5 of glucose, which would not be expected to be significantly impacted by pentose cycling.

The isotope balance equation of the [1-^13^C]oxaloacetate pool is given by Eq. (3):

3 $$	\frac{{\partial \left[ {1 - {}^{13}C} \right]OAA}}{{\partial t}} = \frac{1}{2} * \left[ {1 - {}^{13}C} \right]pyruvate \\ 	* {V_{PC}} + \frac{1}{2} * \left( {\left[ {3 - {}^{13}C} \right]OAA + \left[ {1 - {}^{13}C} \right]acetylCoA} \right) \\ 	* {V_{CS}} - \left[ {1 - {}^{13}C} \right]OAA * \left( {{V_{PC}} + {V_{CS}}} \right).$$

Equation (3) may be simplified with the following assumptions as described^38^:

i) Metabolic and isotopic steady state.
ii) [1-^13^C]pyruvate ~ 0.
iii) [1-^13^C]acetyl-CoA ~ 0.
iv) Complete scrambling of enrichment between [2-^13^C]OAA and [3-^13^C]OAA, as well as between [1-^13^C]OAA and [4-^13^C]OAA by fumarase.

The first assumption was tested by confirming the stability of the glucose labeling in the plasma at the time of sampling (Supplementary Figs. 2b and 3a). The absence of significant label in bicarbonate (which we found to be 1.5 ± 0.1% in rats infused with 40 μmol/(kg-min) of [3-^13^C] lactate (plasma lactate enrichment ~45%)) supports assumptions (ii) and (iii). Assumption (iv) was tested by confirming equal enrichment in C2 and C3 glutamate (Supplementary Table 2).

With these assumptions the equation reduces to Eq. (4)

4 $$	\frac{{\partial \left[ {1 - {}^{13}C} \right]OAA}}{{\partial t}} = \frac{1}{2} * \left( {\left[ {3 - {}^{13}C} \right]OAA * {V_{CS}}} \right) \\ 	- \left[ {1 - {}^{13}C} \right]OAA * \left( {{V_{PC}} + {V_{CS}}} \right).$$

At steady-state, $\frac{{\partial \left[ {1 - {}^{13}C} \right]OAA}}{{\partial t}} = 0.$ Rearranging yields Eq. (5)

5 $$\frac{{{V_{PC}}}}{{{V_{CS}}}} = \left( {\frac{{\left[ {3 - {}^{13}C} \right]OAA}}{{2 * \left[ {1 - {}^{13}C} \right]OAA}}} \right) - 1.$$

Substitution of the fractional ^13^C enrichment of C6 glucose for C3 OAA and the fractional enrichment of C4 glucose for C1 OAA into Eq. (5) yields Eq. (6):

6 $$\frac{{{V_{PC}}}}{{{V_{CS}}}} = \left( {\frac{{\left[ {6 - {}^{13}C} \right]glucose}}{{2 * \left( {\left[ {4 - {}^{13}C} \right]glucose} \right)}}} \right) - 1.$$

To avoid any potential impact of glyceroneogenesis^39, 40^ from [3-^13^C]lactate, which would label C1 and C6 glucose, and based on assumption (iv), we further modified Eq. (6) to generate Eq. (7):

7 $$\frac{{{V_{PC}}}}{{{V_{CS}}}} = \left( {\frac{{\left[ {5 - {}^{13}C} \right]glucose}}{{2 * \left( {\left[ {4 - {}^{13}C} \right]glucose} \right)}}} \right) - 1.$$

### Expanded derivation of *V*_PC_/*V*_CS_

Below we present a complete derivation to assess the potential effect of pyruvate recycling and other minor fluxes we did not include in our (*V*_PC_/*V*_CS_) calculations:

1. Dilution of labeling at the level of α-ketoglutarate (via unlabeled glutamine entering the glutamate pool and exchanging).
2. Production of labeled [1-^13^C]pyruvate due to futile cycling between OAA and pyruvate through ME (*V*_ME_) or the combined action of PEP carboxykinase and PK.
3. Flow of unlabeled mass into the TCA cycle at the level of succinate (*V*_suc_).

If pyruvate cycling is included there will be significant ^13^C enrichment in [1-^13^C]pyruvate, [2-^13^C]pyruvate, and [1-^13^C]acetyl-CoA. The isotope balance equation of [1-^13^C]OAA is given by Eq. (8)

8 $$	\frac{{\delta \left[ {1 - {}^{13}C} \right]OAA}}{{\delta t}} = \frac{1}{2} * \left[ {1 - {}^{13}C} \right]pyruvate \\ 	* {V_{PC}} + \frac{1}{2} * \left( {\left[ {3 - {}^{13}C} \right]OAA + [1 - {}^{13}C]acetyl - CoA} \right) \\ 	* \left( {{V_{suc}} + {V_{CS}}} \right)-\left[ {1 - {}^{13}C} \right]OAA * \left( {{V_{PC}} + {V_{CS}} + {V_{Suc}}} \right).$$

Under conditions where a substrate such as propionate that enters the TCA cycle at the level of succinate is not given *V*_CS_ ~ *V*_CS_ + V_Suc_ so that Eq. (8) can be simplified to Eq. (9):

9 $$	\frac{{\delta \left[ {1 - {}^{13}C} \right]OAA}}{{\delta t}} = \frac{1}{2} * \left[ {1 - {}^{13}C} \right]pyruvate * {V_{PC}} + \frac{1}{2} \\ 	* \left( {\left[ {3 - {}^{13}C} \right]OAA + \left[ {1 - {}^{13}C} \right]acetyl - CoA} \right) \\ 	* {V_{CS}}-\left[ {1 - {}^{13}C} \right]OAA * \left( {{V_{PC}} + {V_{CS}}} \right).$$

### Relationship between α-ketoglutarate and OAA labeling

Dilution of label at the level of α-ketoglutarate due to unlabeled glutamine entering glutamate and exchanging with α-ketoglutarate through AAT is given by Eq. (10):

10 $$K1 = \frac{{\left[ {2 - {}^{13}C} \right]\alpha KG}}{{\left[ {3 - {}^{13}C} \right]OAA}}.$$

### Relationship between [1-^13^C]acetyl-CoA and [2-^13^C]pyuvate labeling

The labeling of Acetyl-CoA relative to pyruvate is reduced by the fraction of acetyl-CoA coming from unlabeled sources such as lipids. On the basis of mass balance this reduction is given by Eq. (11)

11 $$	\left[ {1 - {}^{13}C} \right]acetyl - CoA = \left[ {2 - {}^{13}C} \right]pyruvate \\ 	* \frac{{{V_{PDH}}}}{{{V_{CS}}}} = \left[ {2 - {}^{13}C} \right]pyruvate * {K_2},$$

where

12 $${K_2} = \frac{{{V_{PDH}}}}{{{V_{CS}}}}.$$

### Relationship between [1-^13^C]OAA and [1-^13^C]pyruvate

[1-^13^C]pyruvate can be created from [1-^13^C]OAA either through ME (*V*_ME_) or the combined actions of PEP carboxykinase and PK (*V*_PK_). We define total pyruvate recycling as given by Eq. (13)

13 $${V_{{\rm{PK + ME}}}} = {V_{{\rm{PK}}}} + {V_{{\rm{ME}}}}.$$

At steady state the isotope balance equation for [1-^13^C]pyruvate is given by Eq. (14):

14 $$	0 = \left[ {1 - {}^{13}C} \right]OAA * {V_{PK + ME}} - \left[ {1 - {}^{13}C} \right]pyruvate \\ 	* \left( {{V_{PK + ME}} + {V_{PDH}} + {V_{PC}} + {V_{Lacr}}} \right),$$

which can be rearranged to Eq. (15)

15 $$\left[ {1 - {}^{13}C} \right]pyruvate = \left[ {1 - {}^{13}C} \right]OAA * \left[ {\frac{{{V_{PK + ME}}}}{{{V_{PK + ME}} + {V_{PC}} + {V_{PDH}} + {V_{Lacr}}}}} \right],$$

in which *V*_Lacr_ denotes the loss of pyruvate from the liver via reverse transport of lactate and alanine.

The ratio in brackets in Eq. (15) can be expressed in terms of a constant *K*_3_ yielding Eq. (16),

16 $$\left[ {1 - {}^{13}C} \right]pyruvate = \left[ {1 - {}^{13}C} \right]OAA * {K_3},$$

where

17 $${K_3} = \frac{{{V_{PK + ME}}}}{{{V_{PK + ME}} + {V_{PC}} + {V_{PDH}} + {V_{Lacr}}}}.$$

### Relationship between [3-^13^C]OAA and [2-^13^C]pyruvate

Labeling in [2-^13^C]pyruvate may also arise from pyruvate recycling as described below. At steady state, the isotopic balance equation for [2-^13^C]pyruvate is given by Eq. (18):

18 $$	0 = \left[ {2 - {}^{13}C} \right]OAA * {V_{{\rm{PK + ME}}}} - \left[ {2 - {}^{13}C} \right]pyruvate \\ 	* \left( {{V_{{\rm{PK + ME}}}} + {V_{{\rm{PC}}}} + {V_{{\rm{PDH}}}} + {V_{{\rm{Lacr}}}}} \right).$$

Rearranging yields Eq. (19),

19 $$	\left[ {2 - {}^{13}C} \right]pyruvate \\ 	= \left[ {2 - {}^{13}C} \right]OAA * \left[ {\frac{{{V_{{\rm{PK + ME}}}}}}{{{V_{{\rm{PK + ME}}}} + {V_{{\rm{PC}}}} + {V_{{\rm{PDH}}}} + {V_{{\rm{Lacr}}}}}}} \right]$$

where V_Lacr_ is the loss of pyruvate from the liver via reverse transport of lactate and alanine.

The ratio in brackets can be expressed in terms of a constant *K*_3_ yielding

20 $$\left[ {2 - {}^{13}C} \right]pyruvate = {K_3} * \left[ {2 - {}^{13}C} \right]OAA.$$

Due to fumarase and ME reversibility we and others have found that [2-^13^C]OAA ~ [3-^13^C]OAA so that Eq. (19), substituting in K_3_ from Eq. (17), can be replaced with Eq. (21)

21 $$\left[ {2 - {}^{13}C} \right]pyruvate = {K_3} * \left[ {3 - {}^{13}C} \right]OAA.$$

### Derivation of the steady-state solution

Substituting for [1-^13^C]pyruvate and [1-^13^C]acetyl-CoA (~0 based on assumption i) in Eq. (3) at steady state using the relations derived above (Eqs. (11) and (15)) gives Eq. (22)

22 $$	0 = \frac{1}{2} * {K_3} * \left[ {1 - {}^{13}C} \right]OAA * {V_{{\rm{PC}}}} + \frac{1}{2} * {K_1} \\ 	* \left( {\left[ {3 - {}^{13}C} \right]OAA + {K_2} * {K_3} * \left[ {3 - {}^{13}C} \right]OAA} \right) \\ 	* {V_{{\rm{CS}}}}-\left[ {1 - {}^{13}C} \right]OAA * \left. {\left( {{V_{{\rm{PC}}}} + {V_{{\rm{CS}}}}} \right)} \right),$$

which can be rearranged to Eq. (23)

23 $$	0 = \left[ {1 - {}^{13}C} \right]OAA * \left( {0.5 * {K_3} * {V_{{\rm{PC}}}} - {V_{{\rm{PC}}}} - {V_{{\rm{CS}}}}} \right) + \frac{1}{2} * {K_1} \\ 	 * \left[ {3 - {}^{13}C} \right]OAA * \left( {1 + {K_2} * {K_3}} \right) * {V_{{\rm{CS}}}}.$$

Dividing by *V*_CS_ yields

24 $$	0 = \left[ {1 - {}^{13}C} \right]OAA * \left[ {\left( {0.5 * {K_3} * \frac{{{V_{{\rm{PC}}}}}}{{{V_{{\rm{CS}}}}}} - \frac{{{V_{{\rm{PC}}}}}}{{{V_{{\rm{CS}}}}}}} \right) - 1} \right] + \frac{1}{2} \\ 	* \left[ {3 - {}^{13}C} \right]OAA * {K_1}\left( {1 + {K_2} * {K_3}} \right),$$

25 $$	0 = \left[ {1 - {}^{13}C} \right]OAA * \left[ {\left( {0.5 * {K_3} - 1} \right) * \frac{{{V_{PC}}}}{{{V_{CS}}}} - 1} \right] + \frac{1}{2} \\ 	* \left[ {3 - {}^{13}C} \right]OAA * {K_1}\left( {1 + {K_2} * {K_3}} \right)$$

On the basis of the published work we expect minimal dilution at α-ketoglutarate under these conditions^1^, so that

26 $${K_1}\sim1.$$

Furthermore based on published work^2–4^ as well as our present results (Supplementary Table 1)

27 $${K_2} = \frac{{{V_{{\rm{PDH}}}}}}{{{V_{{\rm{CS}}}}}} < 0.1,$$

28 $${K_3} = \frac{{{V_{{\rm{PK + ME}}}}}}{{{V_{{\rm{PK + ME}}}} + {V_{{\rm{PDH}}}} + {V_{{\rm{PC}}}} + {V_{{\rm{Lacr}}}}}} < 0.1.$$

Substituting *K*_1_ = 1 and setting the *K*_2_*K*_3_ term to ~0 yields Eq. (29):

29 $$0 = \left[ {1 - {}^{13}C} \right]OAA * \left[ {\left( {0.5 * {K_3} - 1} \right) * \frac{{{V_{{\rm{PC}}}}}}{{{V_{{\rm{CS}}}}}} - 1} \right] + \frac{1}{2} * \left[ {3 - {}^{13}C} \right]OAA,$$

which can be rearranged to

30 $$\frac{{{V_{{{PC}}}}}}{{{V_{{{CS}}}}}} = \left[ 0.5* {\frac{{\left[ {3 - {}^{13}C} \right]OAA}}{{\left[ {1 - {}^{13}C} \right]OAA}} - 1} \right] * \frac{1}{{1 - 0.5 * {K_3}}}.$$

Note that based on the experimental measurements of *V*_PK+ME_ under these conditions (Supplementary Table 1) we anticipate a 5% or less impact of pyruvate recycling on the calculated (*V*_PC_/*V*_CS_) ratio. In the maximum case where *V*_PC_ = *V*_PK+ME_ (no gluconeogenesis, just pyruvate recycling) *K*_3_ = 1 and *V*_PC_/*V*_CS_ will be underestimated by a factor of 2.

### Calculation of *V*_PC_/*V*_EGP_

Endogenous glucose production (EGP) is the sum of gluconeogenesis (GNG) and glycogenolysis (GlyNG). The fractional contribution of GNG to EGP can be determined by an infusion of [3-^13^C]lactate to label the triose pool, and the subsequent synthesis and Mass Isotopomer Distribution Analysis (MIDA) of plasma ^13^C-glucose^41^. MIDA analysis is based on the probability of unlabeled (T), and singly labeled triose phosphates (T*) combining to form unlabeled glucose (G0 from TT), singly labeled glucose (G1 from TT* and T*T), and doubly labeled glucose molecule (G2 from T*T*).

Since, the sum of the unlabeled, G0, and labeled, G1 and G2, glucose isotopomers is equal to 1, the distribution of the combination of T and T* can be described by the binomial relationship:

31 $$1 = {{\rm{T}}^2} + 2\left( {{\rm{T}}^*{\rm{T}}} \right) + {\left( {{\rm{T}}^*} \right)^2}.$$

This can also be expressed in terms of the *p*, the probability of triose phosphate enriched with ^13^C above natural abundance, and *q* = (1 − *p*), the probability of triose phosphate that is not enriched above natural abundance.

As glucose is synthesized from combining two trioses with enrichments of *p* and *q*, the isotopomer distribution of glucose (*G*0, *G*1 and *G*2) is

32 $${\left( {p + q} \right)^2} = {p^2} + 2pq + {q^2},$$

where G2 = *p*^2^, G1 = 2*pq* and G0 = *q*^2^.

From the mass spectrum, we can determine the ratio of *G*2/*G*1 and from above, we have

33 $${\rm G}2{\rm{/}}{\rm G}1 = {p^2}{\rm{/}}2pq.$$

Substituting (1 − *p*) for *q*, we have

34 $${\rm G}2{\rm{/}}{\rm G}1 = {p^2}{\rm{/}}2p\left( {1 - p} \right) = p{\rm{/}}2\left( {1 - p} \right).$$

Solving for *p*, or XFE, the fractional triose enrichment, in terms of the ratio of the enrichments of doubly labeled ^13^C-glucose arising from the condensation of two-singly labeled trioses, G2, and singly labeled ^13^C-glucose, G1, we have

35 $$p = {\rm{XFE}} = 1{\rm{/}}\left( {\left( {1 + \left( {1{\rm{/}}\left( {{\rm{2}}{\rm G}{\rm{2/}}{\rm G}{\rm{1}}} \right)} \right)} \right)} \right..$$

The contribution of pyruvate carboxylase flux to EGP, *V*_PC_/*V*_EGP_, is then:

36 $${{\rm{V}}_{{\rm{PC}}}}{\rm{/}}{V_{{\rm{EGP}}}} = {\rm{G2/XF}}{{\rm{E}}^2}$$

The key assumptions are:

i. A binomial probability analysis describes the distribution of ^13^C-glucose isotopomers resulting from the synthesis of two ^13^C-labeled trioses.
ii. One gluconeogenic precursor pool enrichment (if not, *V*_PC_/*V*_EGP_ is underestimated).
iii. Triose phosphates are equilibrated (if not, *V*_PC_/*V*_EGP_ is overestimated).
iv. Using [^13^C]lactate as the tracer to label the triose pool does not include the contribution of glycerol to gluconeogenesis, and hence represents *V*_PC_/*V*_EGP_, as validated in Fig. 2.

In addition to these assumptions, the MIDA calculation defines G2 glucose (enriched with 2-^13^C atoms) as originating from the condensation of 2 trioses, each labeled with a single ^13^C-atom. However, the use of ^13^C-lactate as the tracer, and its subsequent passage through the TCA cycle (with entry through PC and PDH) will lead to trioses containing two ^13^C atoms. Therefore in addition to determining the total *m* + 1 and *m* + 2 enrichment in glucose (GC-MS:CI of glucose pentaacetate, *m/z* 331− > 338), enrichment in the glucose C4-C5-C6 fragment was determined by GC-MS analysis in the EI mode of the glucose aldonitrile pentapropionate derivative by monitoring *m/z* 259− > 265^42^. We corrected for any C2 glucose synthesized from ^13^C_2_-trioses by analysis of the enrichment in the glucose C4C5C6 fragment according to the following equation:

37 $$	{\rm Corrected}\,\left[ {m + 2} \right]\,{\rm glucose} = {\rm Measured}\,\left[ {m + 2} \right]\,{\rm glucose} \\ 	 - 2 * C4C5C6\,\left[ {m + 2} \right]\,{\rm glucose}.$$

### Flux modeling: ex vivo NMR^1,10^ of hepatic tissue

Our flux calculations employ the following equations (Table 3).

**Table 3:** Flux modeling ratios used in the ex vivo NMR method

**Ratio**	**Calculation**
$\frac{{{V_{{\rm{PC}}}}}}{{{V_{{\rm{EGP}}}}}}$	$\frac{{C5\,glucose}}{{C2\,malate}}$ (38)
$\frac{{{V_{{\rm{PC}}}}}}{{{V_{{\rm{CS}}}}}}$	$\frac{{\frac{1}{2} * \left( {C2\,malate + C3\,malate} \right) - C4\,glutamate}}{{C3\,alanine - \left( {C2\,malate + C3\,malate} \right)}}$ (39)

### Absolute *V*_PC_ and *V*_CS_ flux rates

In rats, in which [3-^3^H]glucose was used as the tracer for glucose turnover, EGP was measured as previously described^43^. Based on our previous study in which we demonstrated that net renal gluconeogenesis is negligible, we assume that all gluconeogenesis occurs in the liver^10^.

Combining the relative flux estimations for $\frac{{{V_{{\rm{PC}}}}}}{{{V_{{\rm{EGP}}}}}}$ and $\frac{{{V_{{\rm{CS}}}}}}{{{V_{{\rm{PC}}}}}}$ described above with rates of endogenous glucose production (*V*_EGP_) yields absolute flux rates for *V*_PC_ and *V*_CS_ as follows (Table 4).

**Table 4:** Calculation of hepatic mitochondrial fluxes (V_PC_, *V*_CS_) with PINTA

**Flux**	**Calculation**
*V*_PC_	${V_{EGP}} * \frac{{{V_{PC}}}}{{{V_{EGP}}}}$ (40)
*V*_CS_	${V_{PC}} * \left( {\frac{{{V_{CS}}}}{{{V_{PC}}}}} \right)$ (41)

### NMR, GC/MS and LC/MS-MS analyses

We used GC/MS to measure *m* + 1 and *m* + 2 [^13^C] glucose enrichment. The total *m* + 1 and *m* + 2 [^13^C] glucose enrichment was measured using a pentaacetate derivative: plasma or liver samples were deproteinized using five volumes of methanol, dried and derivatized with 75 μl of 1:1 acetic anhydride:pyridine. After heating to 65 °C for 20 min, the reaction was terminated by adding 25 μl methanol, and *m* + 1 and *m* + 2 (as well as *m* + 3…*m* + 6) were determined by GC/MS (CI mode, *m/z* 331 [*m*], 332 [*m* + 1], 333 [*m* + 2],…337 [*m* + 6]). The *m* + 1 and m + 2 [^13^C] enrichments of the glucose C4C5C6 fragment were determined by generating the aldonitrile pentapropionate derivative: plasma or NMR liver extract samples were dried under N_2_ gas, and 50 μl hydroxylamine hydrochloride (20 mg/ml in pyridine) were added. The samples were heated at 90 °C for 60 min, then 100 μl propionic anhydride was added, after which the samples were heated at 60 °C for 30 min. Finally the samples were evaporated under N_2_ gas, resuspended in ethyl acetate, and the *m* + 1 and *m* + 2 enrichment of the glucose C4C5C6 fragment was measured by GC/MS^42^.

[^13^C]malate enrichment was also measured by GC/MS. A 50–100 mg liver tissue sample was weighed in a 2.0 ml microcentrifuge tube with a metal bead, and 1.0 ml of pre-chilled methanol/water (50:50, v/v) solution was added, followed by disruption at 30 Hz for 1.0 min (Qiagen Tissue Lyser, Valencia, CA) and centrifugation (4,000 r.p.m.) at 4 °C for 10 min. The supernatant was dried in a Speed Vac, and 75 μl *n*-butanol 4 N HCl was added into each residual sample. The resulting mixtures were heated for 30 min at 65 °C. The solvent was removed by a steady-flow of nitrogen gas at room temperature and the dried samples were acetylated with 100 μl trifluoroacetic anhydride in methylene chloride (1:7 v/v) solution. The total and C2 + C3 malate enrichments were determined by GC/MS (EI mode, *m/z* 213 for the total and 186 for the C1C2C3 fragment). The C2 + C3 malate enrichment was determined according to Eq. (42), which relies on the assumption that the C4 enrichment is approximately equal to the C1 enrichment of malate

42 $${\rm{AP}}{{\rm{E}}_{{\rm{C2C3}}}} = {\rm{AP}}{{\rm{E}}_{{\rm{total}}}}-2 * \left( {{\rm{AP}}{{\rm{E}}_{{\rm{total}}}}-{\rm{AP}}{{\rm{E}}_{{\rm{C1C2C3}}}}} \right).$$

[^13^C]alanine enrichment was measured by GC/MS as we have described^7^. [^13^C] NMR spectroscopy using the Bruker Topspin system was utilized to measure relative positional ^13^C-enrichment of glucose^7^, and combined with the total [^13^C] glucose enrichment ([*m* + 1] + 2 * [*m* + 2]) measured by GC/MS to calculate the positional enrichment of [^13^C] glucose.

Total [^13^C]glutamate enrichment was determined by LC-MS/MS. Approximately 100 mg of liver tissue was weighed out and homogenized in 500 μl ice-cold methanol using a TissueLyser, then centrifuged at 4,000 r.p.m. for 10 min. The supernatant was then purified by centrifugation (4,000 r.p.m., 10 min) through the Pall Nanosep filter (100 K) (Port Washington, NY). Glutamate ^13^C enrichment ([*m* + 1], [*m* + 2]) was then assessed by LC-MS/MS using an AB Sciex QTRAP 6500 (Framingham, MA), equipped with a Shimadzu ultra-fast liquid chromatography (UFLC) system (Columbia, MD) and electrospray ionization (ESI) source with negative-ion detection. The measured ion pairs were 146/128 (m0), 147/129 (*m* + 1) and 148/130 (*m* + 2).

Positional enrichment of glucose, glutamate, and alanine was measured by ^13^C NMR. The samples were run on the AVANCE 500-MHz NMR spectrometer (Bruker Instruments, Billerica, MA). Spectra were acquired with relaxation time = 1 s, dummy scans = 4, and number of scans = 25,000. Correction factors for differences in T_1_ relaxation times were determined from fully relaxed spectra of standard glucose, glutamate and alanine solutions. The total enrichment measured by GC/MS (glucose and alanine) or LC-MS/MS (glutamate) was divided algebraically between the carbons of each molecule based on the relative areas of the ^13^C NMR peaks: the NMR fraction of the total label in each carbon was calculated, and then multiplied by the total enrichment ([*m* + 1] APE + 2*[*m* + 2] APE + 1.1% APE * *n*, where *n* is the number of carbons in the molecule of interest). The chemical shifts of each peak are shown in Table 5.

**Table 5:** Chemical shifts of peaks examined by ^13^C NMR

**Peak**	**Chemical shift (p.p.m.)**
α − C1 glucose	92.3
β − C1 glucose	96.8
α − C2,α − C5 glucose	72.5
β − C2 glucose	75.0
α − C3 glucose	73.6
β − C3 glucose	76.6
α,β − C4 glucose	70.5
β − C5 glucose	76.8
α,β − C6 glucose	61.5
C1 glutamate	175.8
C2 glutamate	55.6
C3 glutamate	28.5
C4 glutamate	34.6
C5 glutamate	182.0
C2 alanine	51.7
C3 alanine	17.3

^13^C enrichment in C1 alanine was assumed to be negligible and the total alanine ^13^C enrichment was divided between carbons 2 and 3.

### Liver glycogen content

Hepatic glycogen concentrations were analyzed following amyloglucosidase digestion and measurement of glucose concentrations in the digest as previously described^44^.

### Statistical analysis

GraphPad Prism version 7.0 was used to perform all statistical analysis. Comparisons of two groups were performed using the two-tailed unpaired Student’s *t*-test, while a simple linear regression was performed to analyze linear correlations. Differences with *P*-value < 0.05 were considered significant. GraphPad Prism software confirmed that the variances were not significantly different between groups compared using a student *t*-test, and a normal distribution was assumed.

### Data availability

The data that support the findings of this study are available from the corresponding author (G.I.S.) upon reasonable request.

## Electronic supplementary material

Supplementary Information
