# Publisher Correction: Non-invasive assessment of hepatic mitochondrial metabolism by positional isotopomer NMR tracer analysis (PINTA)

**DOI:** 10.1038/s41467-018-03023-3

**Published:** 2018-01-31

**Authors:** Rachel J. Perry, Liang Peng, Gary W. Cline, Gina M. Butrico, Yongliang Wang, Xian-Man Zhang, Douglas L. Rothman, Kitt Falk Petersen, Gerald I. Shulman

**Affiliations:** 1 Departments of Internal Medicine, Yale University School of Medicine, New Haven, CT 06520 USA; 2 Departments of Radiology and Biomedical Imaging, Yale University School of Medicine, New Haven, CT 06520 USA; 3 Departments of Biomedical Engineering, Yale University School of Medicine, New Haven, CT 06520 USA; 4 Departments of Cellular and Molecular Physiology, Yale University School of Medicine, New Haven, CT 06520 USA; 5 Departments of Howard Hughes Medical Institute, Yale University School of Medicine, New Haven, CT 06520 USA

Correction to: *Nature Communications* 10.1038/s41467-017-01143-w, published online: 06 Oct 2017

The originally published version of this Article contained an error in Equation 30, which was inadvertently introduced during the production process. The incorrect form of Equation 30 was:

$$\frac{{V_{PC}}}{{V_{CS}}} = \left( {\left[ {\frac{{[3 - {}^{13}C]OAA}}{{[1 - {}^{13}C]OAA}} - 1} \right] \ast \frac{1}{{1 - 0.5 \ast K_3}} \ast \frac{1}{2}} \right)$$

The correct form of Equation 30 is:

$$\frac{{V_{PC}}}{{V_{CS}}} = \left[ {0.5 \ast \frac{{[3 - {}^{13}C]OAA}}{{[1 - {}^{13}C]OAA}} - 1} \right]\ast \frac{1}{{1 - 0.5 \ast K_3}}$$

This has now been corrected in the PDF and HTML versions of the Article.

