# Supplementary material for: Non-invasive assessment of hepatic mitochondrial metabolism by positional isotopomer NMR tracer analysis (PINTA)
Source: Nat Commun. 2017 Oct 6;8:798. doi: 10.1038/s41467-017-01143-w (PMC5630596; doi:10.1038/s41467-017-01143-w)
Supplement: Supplementary file 1 — Supplementary Information [file 41467_2017_1143_MOESM1_ESM.pdf]

	[1-^{13}C] Glucose	[2-^{13}C] Glucose	[3-^{13}C] Glucose	[4-^{13}C] Glucose	[5-^{13}C] Glucose	[6-^{13}C] Glucose	[2-^{13}C] Glutamate	[3-^{13}C] Glutamate	V_{PDH}/ V_{CS} (%)	V_{PK+ME}/ V_{PC+PDH} (%)
Control	6.0±0.3	4.6±0.2	0.9±0.1	0.9±0.1	5.7±0.3	7.8±0.4	7.3±0.4	7.1±0.4	3.4±0.1	5.9±0.1
ME inhibitor	3.6±0.5	2.7±0.3	0.6±0.1	0.6±0.1	3.3±0.4	4.5±0.5	4.4±0.7	4.5±0.6	2.0±1.1	7.9±2.1
Glucagon	4.9±0.4	3.5±0.2	1.3±0.2	1.2±0.1	4.0±0.3	6.0±0.4	4.6±0.5	4.8±0.6	2.7±0.1	<2%
Epinephrine	5.0±0.3	3.9±0.2	0.4±0.1	0.5±0.1	5.1±0.4	6.8±0.4	5.5±0.4	5.5±0.2	4.3±1.0	5.4±0.1
Hyperinsulinemic-euglycemic clamp	3.8±0.4	2.9±0.3	0.6±0.2	0.8±0.1	3.7±0.4	4.9±0.5	5.4±0.5	5.3±0.5	3.8±0.5	14.8±4.6
HFD	4.1±0.3	3.0±0.3	0.9±0.2	0.7±0.2	3.2±0.3	4.4±0.7	4.8±0.6	4.8±0.4	2.6±0.4	6.6±1.2
HFD-CRMP	3.4±0.8	2.5±0.7	1.0±0.2	1.0±0.2	3.4±0.9	4.2±1.0	4.6±0.3	4.5±0.4	4.1±0.5	6.1±0.6
**Average (all groups)**									**3.8±0.5**	**5.8±0.7**

Supplementary Table 1. Raw data from all groups of rats in this study. Data are presented as the mean±S.E.M. of n=16 (controls), n=5 (HFD-CRMP), or n=6 (all other groups) rats per group.

	Total [m+1] Glucose	Total [m+2] Glucose	C4C5C6 [m+2] Glucose	V_{PC}/V_{EGP} (%)
Control	26.9±1.0	5.7±0.5	0.42±0.06	72±2
ME inhibitor	15.2±1.7	2.1±0.6	0.07±0.04	50±2
Glucagon	22.3±2.2	4.2±0.7	0.24±0.06	65±3
Epinephrine	22.2±1.6	4.0±0.8	0.25±0.05	67±2
Hyperinsulinemic-euglycemic clamp	11.2±3.1	1.6±0.7	0.16±0.03	42±6
HFD	24.0±1.5	5.3±0.6	0.64±0.22	64±5
HFD-CRMP	20.0±4.2	3.9±1.4	0.23±0.10	58±7

Supplementary Table 2. Glucose enrichment in all groups of rats in this study. Data are presented as the mean±S.E.M. of n=16 (controls), n=5 (HFD-CRMP), or n=6 (all other groups) rats per group.

Supplementary Figure 1

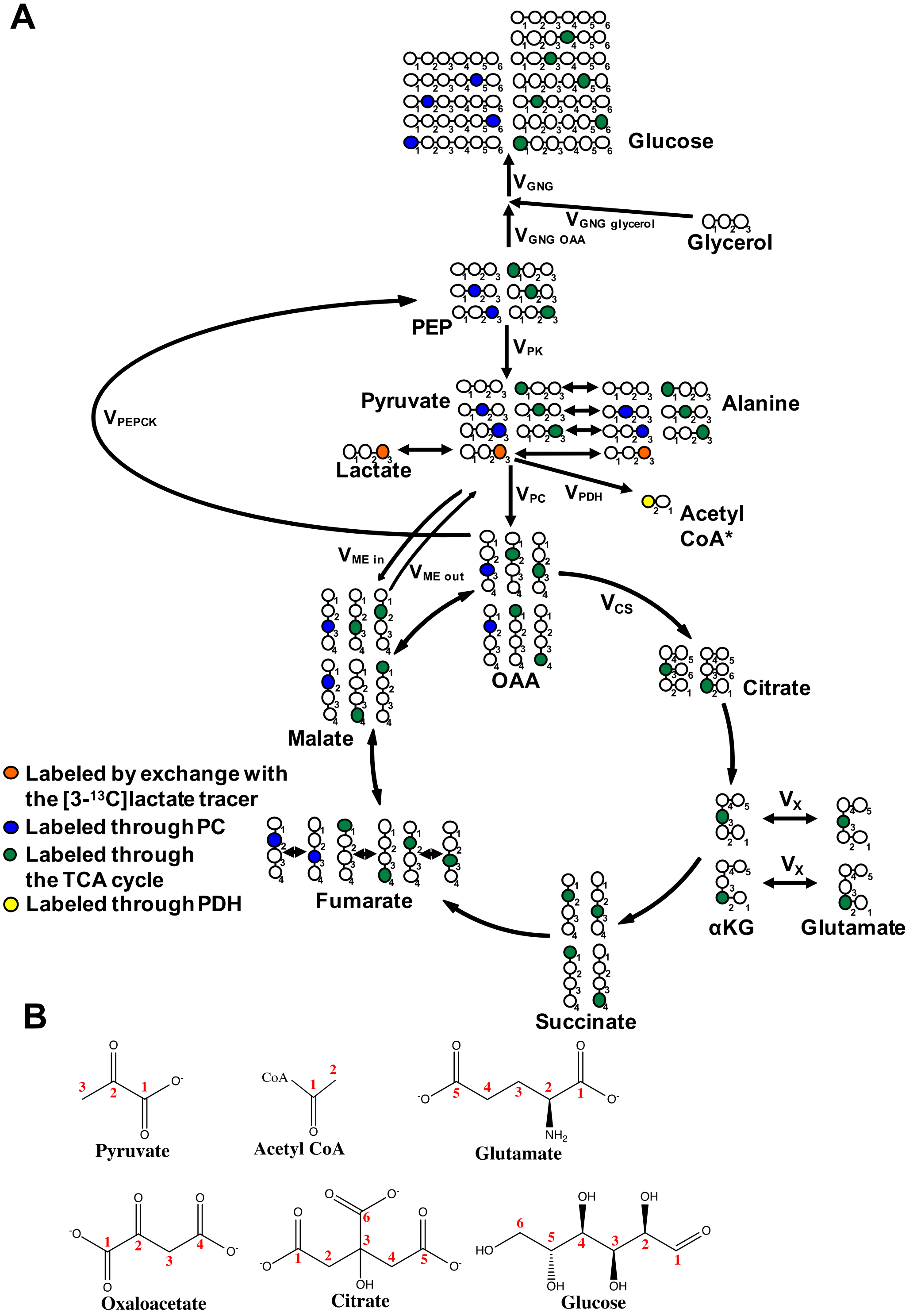

Supplementary Fig. 1. (A) [3-^{13}C]lactate tracer labeling scheme. αKG, α-ketoglutarate; CS, citrate synthase; GNG, gluconeogenesis; ME, malic enzyme; OAA, oxaloacetate; PEPCK, phosphoenolpyruvate carboxykinase. (B) Chemical structures of key metabolites with the labeling convention used in this study.

Supplementary Figure 2

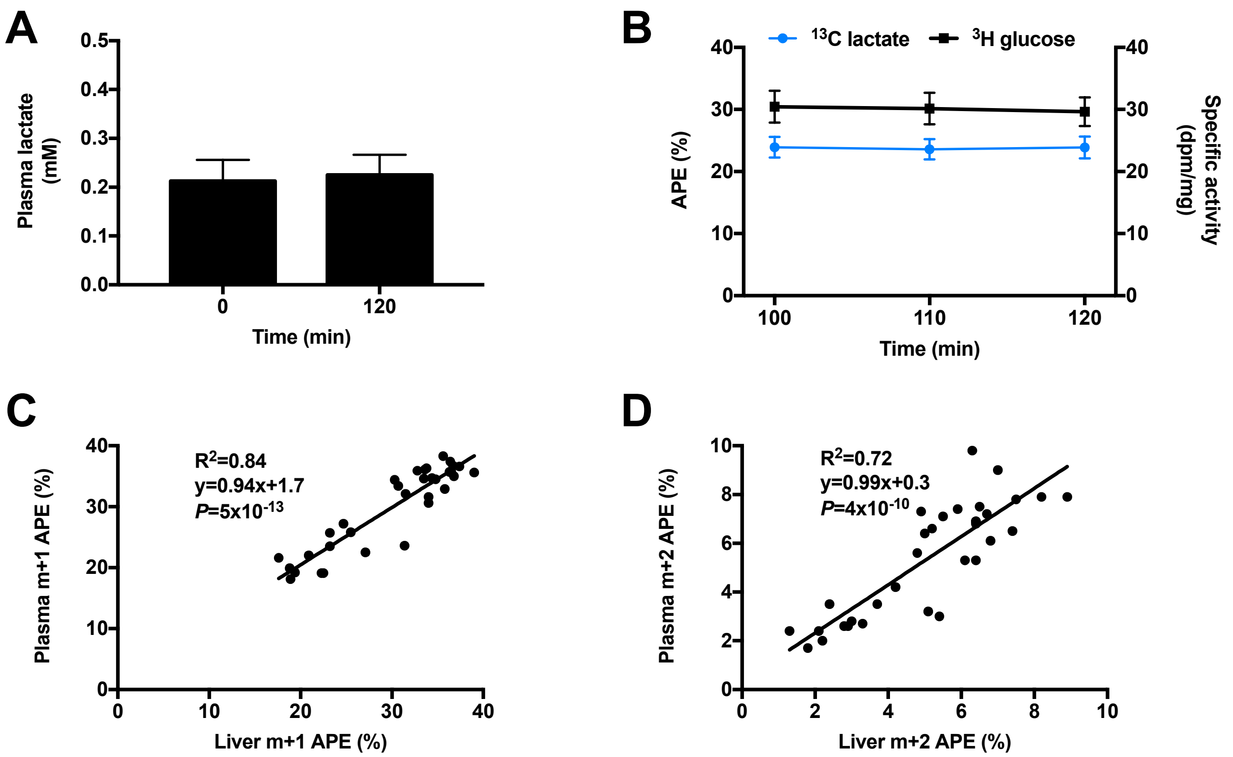

Supplementary Fig. 2. Plasma glucose enrichment is a good surrogate for liver glucose enrichment in rats infused with [3-^{13}C]lactate at steady-state. (A) Plasma lactate concentrations before and after a 120 min infusion of [3-^{13}C]lactate. In panels (A) and (B), data are the mean ± S.E.M. of n=16 per time point. (B) Plasma ^{13}C lactate enrichment and ^{3}H glucose specific activity at steady-state. (C) m+1 glucose enrichment. (D) m+2 glucose enrichment.

Supplementary Figure 3

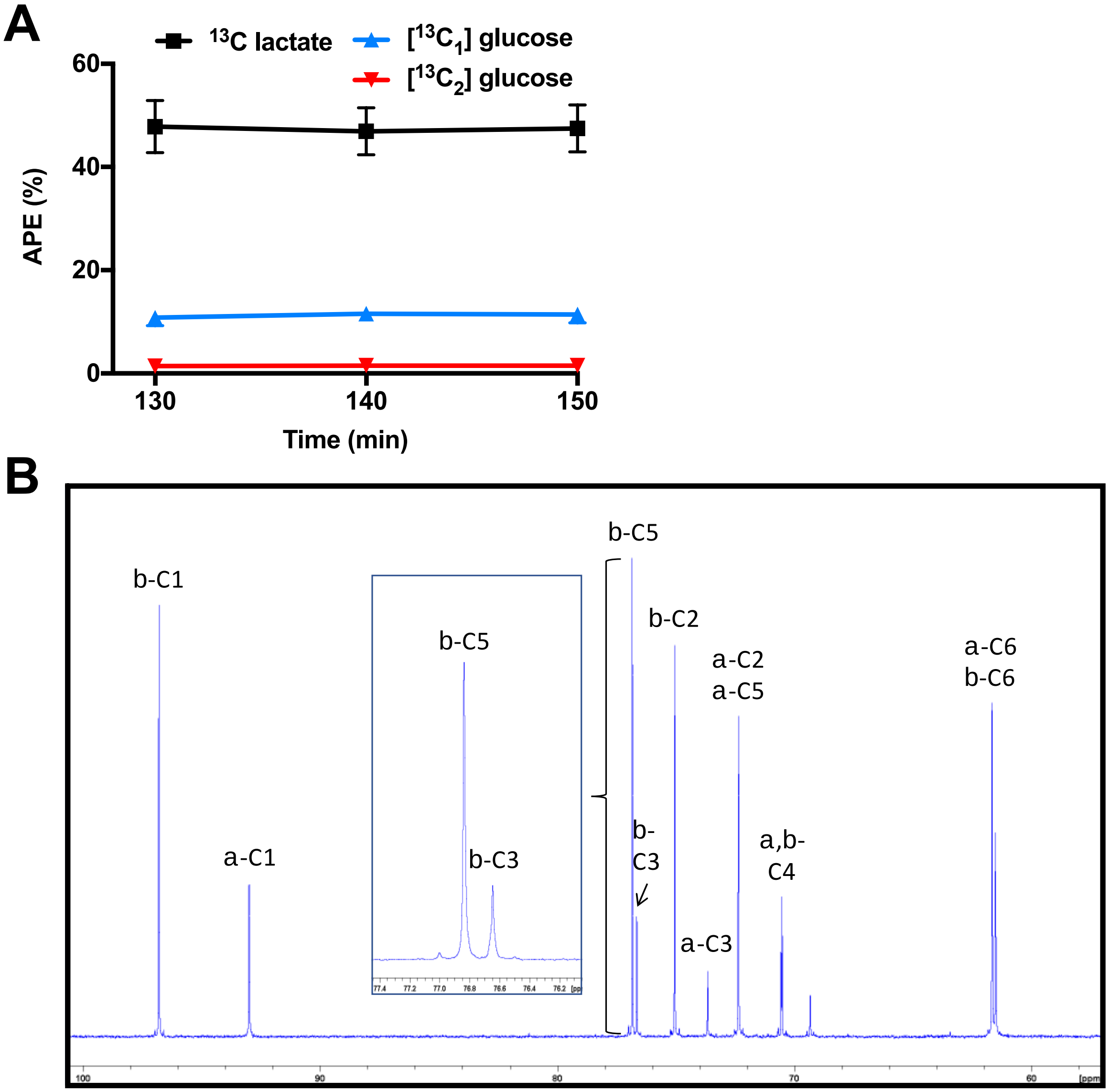

Supplementary Fig. 3. Calculation of hepatic fluxes in non-diabetic, overnight fasted humans. (A) Plasma [^{13}C]lactate and m+1 and m+2 [^{13}C]glucose enrichments. Data are the mean ± S.E.M. of three subjects. (B) Representative ^{13}C NMR spectrum of plasma glucose.

Supplementary Figure 4

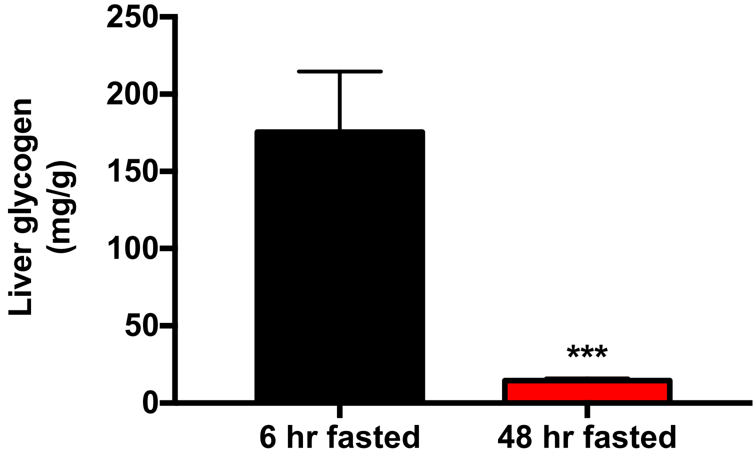

Supplementary Fig. 4. Hepatic glycogen content is reduced after a 48 hr fast. Data are the mean±S.E.M. of n=5 per group.
